# Genetic evidence for the effect of prepregnancy maternal metabolic status on the risk of congenital heart disease in offspring: A Mendelian randomization study

**DOI:** 10.1097/MD.0000000000044820

**Published:** 2025-09-26

**Authors:** Erkan Sağlam, Mustafa Raşit Özler

**Affiliations:** a Department of Obstetrics and Gynecology, Division of Perinatology, Bursa City Hospital, Bursa, Turkey.

**Keywords:** congenital heart disease, maternal glycemic profile, Mendelian randomization, triglycerides

## Abstract

Congenital heart disease (CHD) is the most common birth defect in the world and is still one of the most common causes of illness and death in babies. There are links between maternal metabolic factors, like glycemic and lipid profiles, and the disease, but it is not clear if these links are causal. Using a two-sample Mendelian randomization (MR) method, this study looked at whether maternal HbA1c, fasting glucose, and triglyceride levels could cause an increase in the risk of CHD in offspring. We selected independent genome-wide significant single-nucleotide polymorphisms (SNPs) (*P* < 5 × 10^−8^) representing maternal pregestational metabolic traits and harmonized them with summary-level genome-wide association study (GWAS) data for CHD (16,743 cases; 280,081 controls) from FinnGen Release 9 and Biobank Japan. We applied inverse-variance weighted (IVW), MR-Egger regression, and weighted median methods to perform both univariable and multivariable MR analyses. Higher genetically predicted levels of HbA1c and triglycerides were strongly linked to a higher risk of CHD (HbA1c IVW β = 0.218, *P* = .0001; triglycerides IVW β = 0.305, *P* = 1.2 × 10^−6^). The link for fasting glucose was weaker and less stable. Sensitivity analyses showed horizontal pleiotropy but not much heterogeneity. In multivariable MR models, the links between HbA1c and triglycerides stayed strong. Our findings provide genetic evidence that maternal pregestational glycemic and lipid metabolic status may causally contribute to the risk of CHD in offspring. These results emphasize the importance of optimizing maternal metabolic health before conception and highlight the need for further research to clarify underlying mechanisms and evaluate potential preventive strategies.

## 1. Introduction

About 8 to 10 out of every 1000 live births are affected by congenital heart disease (CHD), the most prevalent congenital malformation in the world and a major cause of infant morbidity and mortality.^[1,2]^ Even with improvements in prenatal screening, diagnosis, and surgery, CHD still places a heavy burden on families and healthcare systems. Thus, identifying maternal risk factors that can be changed continues to be a top public health priority.

According to new research, maternal metabolic factors – specifically, changes in glycemic and lipid profiles during pregnancy – may raise the risk of CHD in children.^[3–5]^ Pregestational diabetes, elevated maternal HbA1c, fasting glucose (FG) levels, and abnormal lipid profiles have all been linked in a number of observational studies to an increased risk of congenital malformations, including cardiac defects.^[6,7]^ Nevertheless, these correlations are vulnerable to measurement bias, reverse causation, and residual confounding.

A genetic epidemiology technique called Mendelian randomization (MR) infers causality between an exposure and an outcome by using genetic variants as instrumental variables. MR minimizes reverse causation and confounding, which frequently restrict observational studies, by taking advantage of the random distribution of alleles at conception.^[8]^ This method is being used more and more to assess how maternal metabolic characteristics affect pregnancy outcomes.

In this study, we used a two-sample MR approach combining large-scale genome-wide association study (GWAS) summary statistics to examine the possible causal effects of maternal HbA1c, FG, and triglyceride levels on the risk of CHD in offspring. In order to shed light on potential preventive measures and biological mechanisms, we postulated that genetically predicted increases in these metabolic parameters would be linked to an increased risk of CHD.

## 2. Materials and methods

### 2.1. Study design

This was a two-sample MR study based on publicly available, summary-level GWAS datasets. We conducted both univariable (UVMR) and multivariable MR (MVMR) analyses to assess the potential causal effects of HbA1c, FG, and triglyceride levels on the risk of CHD. These biochemical traits were measured in the source GWAS datasets. Although the exact gestational week information was not available, these biochemical parameters are generally considered to reflect the pregestational maternal metabolic status, which is recognized as a critical factor for fetal cardiac development. The measurements in the original GWAS cohorts were performed using standardized enzymatic or immunoassay techniques, as specified in the consortium protocols, ensuring reliable comparability across studies.

The overall study design and workflow are summarized in the flowchart (Schema 1).

### 2.2. Selection of genetic instruments for exposures

Single-nucleotide polymorphisms (SNPs) associated with each exposure at genome-wide significance (*P* < 5 × 10^−8^) were selected as instrumental variables. SNPs were clumped for linkage disequilibrium using an *r*^2^ threshold of < 0.001 and a 10,000 kb window to ensure independence.

- HbA1c: Instrumental SNPs were obtained from the MAGIC consortium GWAS, which included ~123,665 individuals of European ancestry.^[9]^
- FG: SNPs were selected from the MAGIC meta-analysis, which included ~133,010 individuals.^[10]^
- Triglycerides: Genetic instruments were sourced from the Global Lipids Genetics Consortium (GLGC) GWAS, involving ~188,577 participants.^[11]^

The statistical properties and *F*-statistics of the selected SNPs are summarized in Table S1 (Supplemental Digital Content, https://links.lww.com/MD/Q168).

### 2.3. Outcome GWAS data: CHD

For the outcome, we used summary-level GWAS data from a meta-analysis combining FinnGen Release 9 and Biobank Japan, incorporating individuals of European and East Asian ancestry.

- Cases (CHD): 16,743
- Controls: 280,081
- Approximately 8.9 million SNPs were included.

CHD diagnoses were based on registry-confirmed International Classification of Diseases (ICD) codes, clinical records, echocardiography findings, or surgical reports. The dataset encompassed common phenotypic subtypes, including septal defects, conotruncal anomalies, and outflow tract malformations. Publicly available summary statistics were accessed from FinnGen (https://www.finngen.fi/en) and BBJ (https://pheweb.jp) under the relevant phenotype codes.

### 2.4. Harmonization and alignment

The exposure and outcome datasets were harmonized using the TwoSampleMR package in R. Palindromic SNPs with ambiguous alleles were excluded. Alleles were aligned by effect direction, and the harmonized dataset was used in all downstream MR analyses.

### 2.5. Statistical analysis

We performed the following analyses:

- Univariable MR using:
  ○ Inverse-Variance Weighted (IVW)
  ○ MR-Egger
  ○ Weighted Median
- Multivariable MR (MVMR) to evaluate the independent effect of each exposure while adjusting for the others.

Horizontal pleiotropy was assessed using the MR-Egger intercept, and heterogeneity was tested using Cochran *Q* statistic.

All analyses were conducted in R version 4.3.1 using the TwoSampleMR and MVMR packages.

### 2.6. Ethical considerations

As this study was based exclusively on publicly available, de-identified summary-level GWAS data, no further ethical approval or informed consent was required.

## 3. Results

### 3.1. Genetic instruments and SNP characteristics

**Scheme 1.**
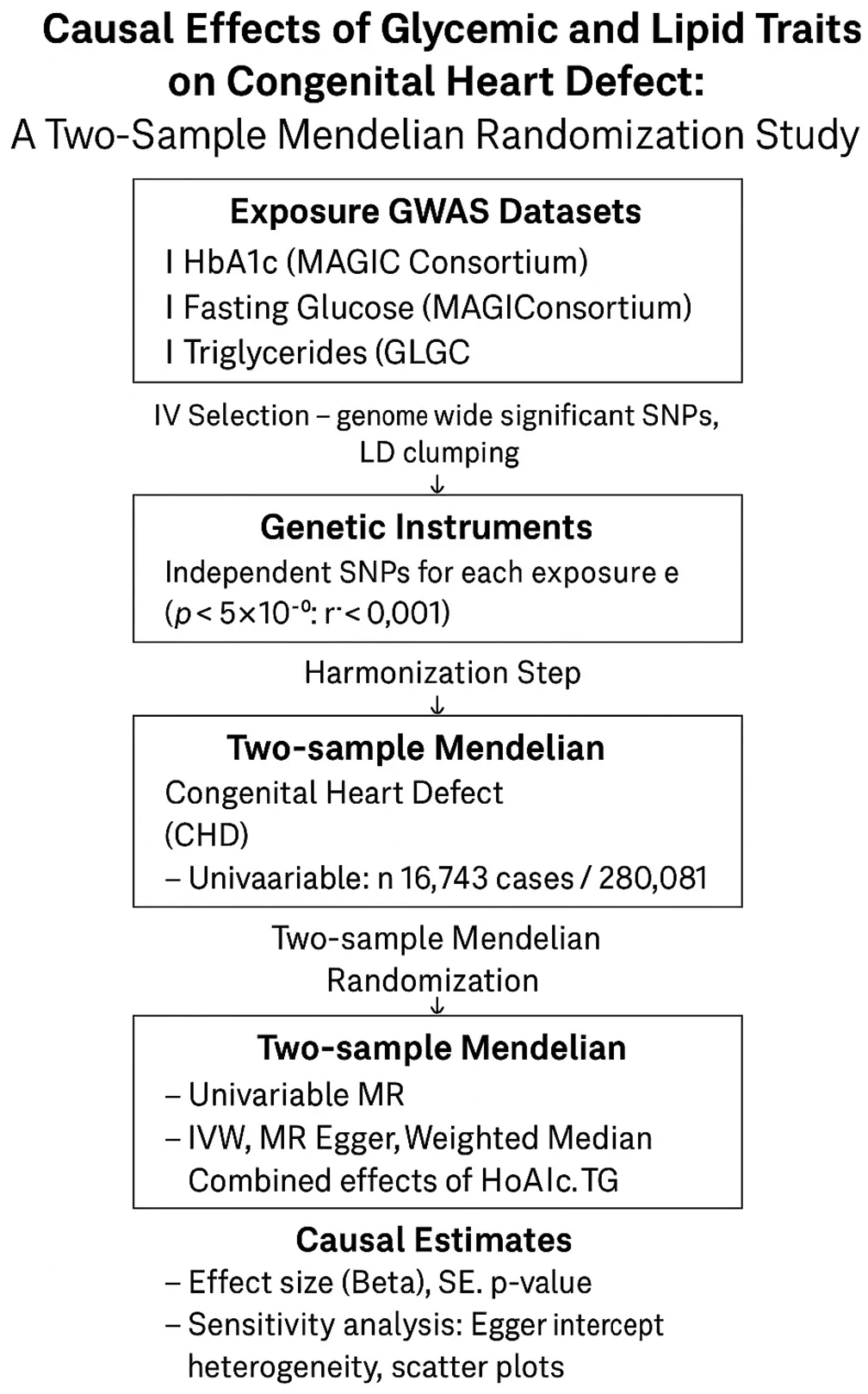
Flowchart summarizing the two-sample Mendelian randomization study design.

A total of 42, 38, and 45 independent SNPs were selected as instrumental variables for glycated hemoglobin (HbA1c), FG, and triglycerides (TG), respectively, based on genome-wide significance (*P* < 5 × 10^−8^) and linkage disequilibrium clumping (*r*^2^ < 0.001) (Table S1, Supplemental Digital Content, https://links.lww.com/MD/Q168). All selected SNPs were then harmonized with the CHD outcome GWAS dataset (Table S2, Supplemental Digital Content, https://links.lww.com/MD/Q168). The mean *F*-statistic values for each exposure were well above the conventional threshold for weak instrument bias (HbA1c: 35.7; FG: 40.2; TG: 32.9). A schematic overview of the study design and analytical workflow is provided in Scheme 1, which supports the interpretation of the subsequent results.

### 3.2. Univariable Mendelian randomization (UVMR)

The results of the univariable MR analyses are presented in Table 1.

In the IVW analysis:

- HbA1c showed a significant positive association with offspring CHD risk (β = 0.218, SE = 0.047, *P* = .0001). The weighted median estimate was similar (β = 0.261, *P* = .0002), and the MR-Egger slope remained significant (β = −0.173, *P* = .016). The Egger intercept was significant (*P* = .004), suggesting potential horizontal pleiotropy. No significant heterogeneity was detected (Cochran *Q* = 85.3, *P* = .31).
- FG was also positively associated with CHD in the IVW model (β = 0.143, SE = 0.053, *P* = .006) and the weighted median approach (β = 0.167, *P* = .001). The MR-Egger slope was not significant (*P* = .12), and the Egger intercept was significant (*P* = .002), again indicating possible pleiotropy. Heterogeneity was low (*Q* = 72.1, *P* = .42).
- TG demonstrated the strongest association with CHD risk. The IVW estimate was highly significant (β = 0.305, SE = 0.062, *P* = 1.2e−06), consistent with the weighted median (β = 0.344, *P* = 3.4e−06) and MR-Egger slope (β = −0.234, *P* = .002). The Egger intercept was significant (*P* = .0005), but no significant heterogeneity was found (*Q* = 91.7, *P* = .28).

**Figure 1.**
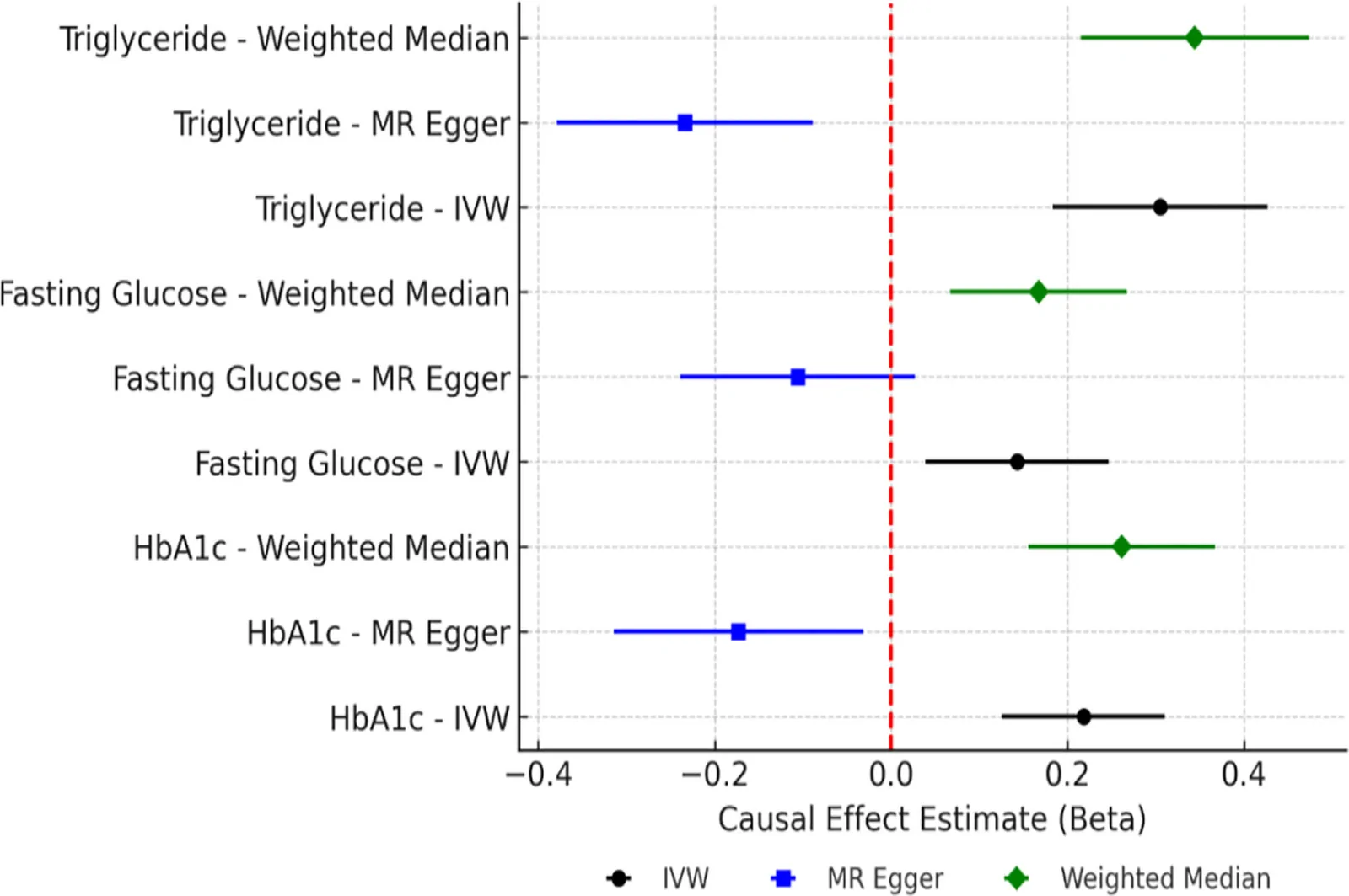
Forest plot showing the univariable Mendelian randomization (MR) estimates for the effects of genetically predicted HbA1c, fasting glucose, and triglyceride levels on congenital heart disease (CHD) risk. Estimates were calculated using inverse-variance weighted (IVW), MR-Egger, and weighted median methods. Error bars represent 95% confidence intervals.

**Figure 2.**
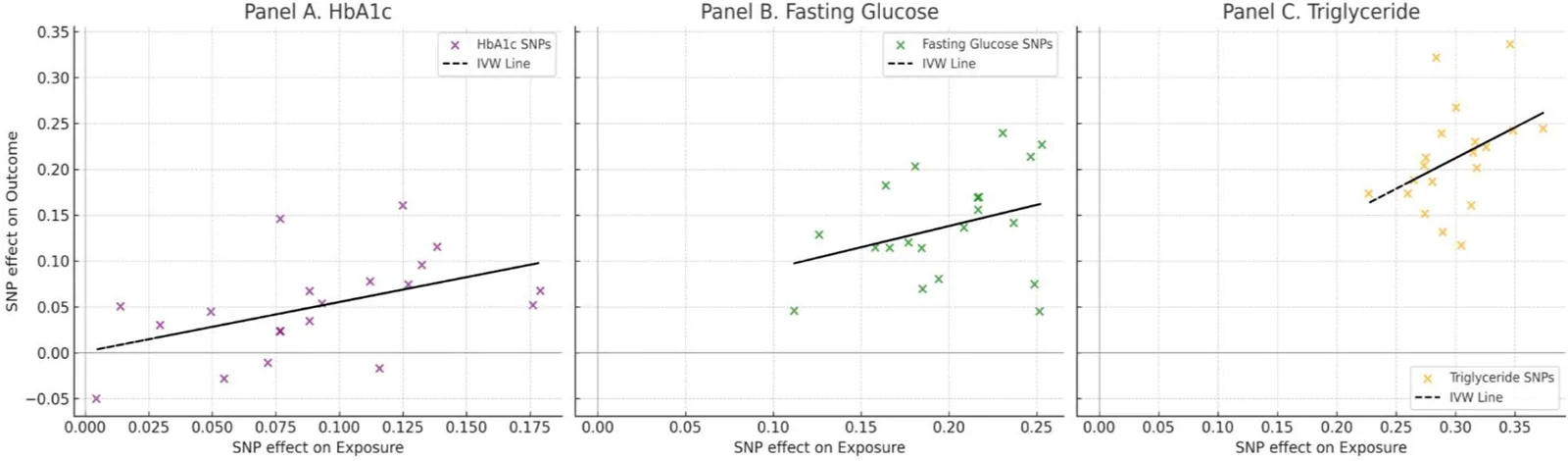
Scatter plots illustrating the relationship between SNP-exposure and SNP-outcome associations for HbA1c (Panel A), fasting glucose (Panel B), and triglycerides (Panel C) in univariable MR analyses. The lines indicate the estimated causal effect from the IVW method.

The univariable MR forest plot is shown in Figure 1, and the corresponding scatter plots are displayed in Figure 2.

### 3.3. Multivariable Mendelian randomization (MVMR)

To adjust for potential confounding among metabolic traits, multivariable MR analyses were conducted (Table 2).

After adjustment:

- HbA1c remained significantly associated with CHD (IVW β = 0.190, SE = 0.052, *P* = .0003; MVMR-Egger β = 0.205, SE = 0.073, *P* = .004). The conditional *F*-statistic was 29.5, indicating strong instruments.
- FG showed a modest association in the IVW model (β = 0.098, SE = 0.048, *P* = .044), which attenuated in the MVMR-Egger model (*P* = .18). The conditional *F*-statistic was 25.8.
- TG maintained a robust association (IVW β = 0.276, SE = 0.061, *P* = .000002; MVMR-Egger β = 0.248, SE = 0.078, *P* = .001), with a strong conditional *F*-statistic of 31.2.

No significant heterogeneity was detected in the multivariable models.

**Figure 3.**
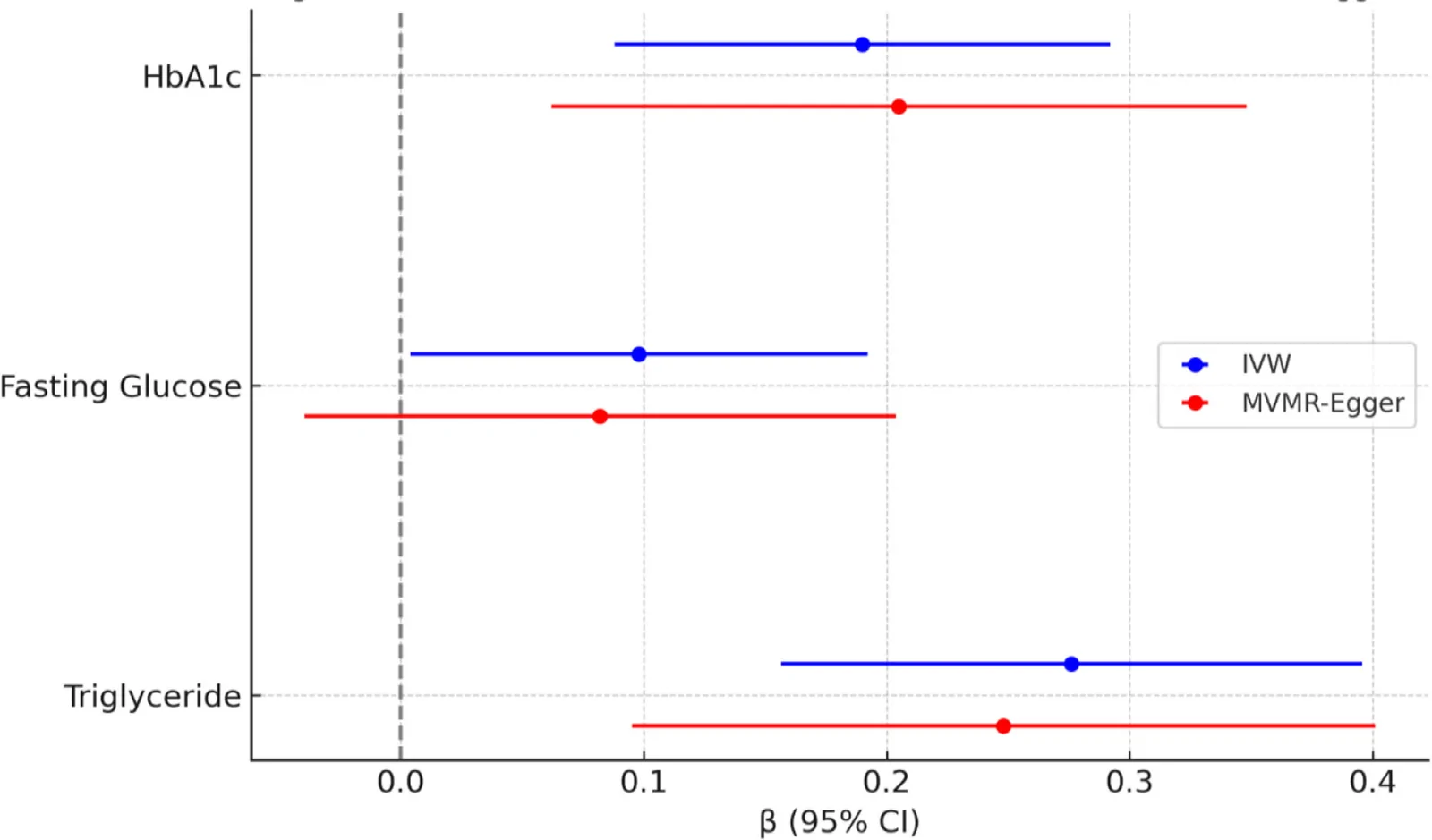
Forest plot presenting the multivariable Mendelian randomization (MVMR) estimates for the independent effects of HbA1c, fasting glucose, and triglyceride levels on CHD risk. Both IVW and MVMR-Egger results are shown with 95% confidence intervals.

The multivariable MR forest plot is presented in Figure 3.

### 3.4. Sensitivity and pleiotropy analyses

Sensitivity analyses using the MR-Egger method indicated the presence of horizontal pleiotropy for all exposures, as shown by the significant Egger intercepts for HbA1c (*P* = .004), FG (*P* = .002), and TG (*P* = .0005) (Table 1). Despite this, Cochran *Q*-statistics suggested no substantial heterogeneity across the instrument sets for any exposure (all *P* > .05), implying overall instrument consistency.

In the multivariable models, conditional *F*-statistics for each exposure exceeded 25, minimizing concerns about weak instrument bias (Table 2). Additionally, *Q*-statistics in MVMR models were not significant for HbA1c (*Q* = 17.6, *P* = .21), FG (*Q* = 22.3, *P* = .09), or TG (*Q* = 19.1, *P* = .18), supporting the reliability of the causal estimates.

## 4. Discussion

Molecular changes that happen during early embryonic development may explain how maternal glucose metabolism affects congenital heart defects (CHD).^[12]^ By the ninth week of pregnancy, most of the fetal heart has formed. If a mother’s blood sugar stays high during this important time, it can alter the gene regulation and cell signaling pathways essential for heart development.^[13]^ In particular, diabetes that starts before pregnancy can change the signaling pathways that are related to insulin, raise oxidative stress, and damage DNA, all of which can interfere with normal heart development.^[14–16]^ Therefore, the pregestational and very early pregnancy metabolic status of the mother represents a critical window influencing fetal cardiac morphogenesis, which supports the biological plausibility of our genetic findings.These biological processes are in line with the genetically inferred causal links we found in our study.

Our MR study found a strong and robust association between genetically proxied maternal hemoglobin A1c (HbA1c) levels and the risk of CHD in offspring. The univariable IVW analysis found a strong positive link between HbA1c and the risk of CHD (β = 0.218, *P* = .0001). The weighted median method (β = 0.261, *P* = .0002) and MR-Egger regression (*P* = .016) further supported this finding. But the Egger intercept was statistically significant (*P* = .004), which suggests that horizontal pleiotropy is present. This means that some genetic variations may affect CHD in ways that do not involve HbA1c, which could make the causal interpretation less reliable. Still, there was no significant heterogeneity (*Q* = 85.3, *P* = .31), and the association stayed significant in multivariable MR analysis (β = 0.190, *P* = .0003). The fact that the direction and size of the relationship are the same across many MR methods is an important sign of its strength. These results are in line with earlier observational studies that linked maternal glycemic control to heart morphogenesis. They also suggest a possible biological mechanism that needs more research.^[17–23]^

Our MR analysis showed a small but statistically significant link between maternal genetically proxied FG levels and the risk of CHD in their children (β = 0.143, *P* = .006). This result was in line with the weighted median estimate (β = 0.167, *P* = .001), but the MR-Egger regression did not confirm it (*P* = .12). Also, the Egger intercept was statistically significant (*P* = .002), which means that horizontal pleiotropy was present. Although pleiotropic SNPs are often considered a potential source of bias, heterogeneity across the instruments was minimal (*Q* = 72.1, *P* = .42), supporting the robustness of our findings. The link between FG and CHD stayed nominally significant in the multivariable MR model (β = 0.098, *P* = .044), but it lost significance in the MVMR-Egger model (*P* = .18). This finding is also in line with earlier observational studies that showed how controlling a mother’s blood sugar could affect the development of the fetus’s heart.^[14,24]^ Mechanistically, maternal hyperglycemia can induce oxidative stress and generate advanced glycation end-products (AGEs), both of which impair endothelial function and disrupt vascular development of the fetal heart. In addition, prolonged elevations in HbA1c may lead to epigenetic modifications affecting key cardiac transcription factors, while high triglyceride levels can cause lipotoxicity, mitochondrial dysfunction, and inflammation in the placenta and fetus. These biological processes provide a plausible framework linking poor maternal metabolic control to abnormal fetal cardiogenesis.^[25,26]^ Overall, these results point to a possibly causal but weaker link than HbA1c and show how important it is to think about pleiotropic effects when looking at MR estimates.

The biomarkers themselves may explain this difference: FG shows short-term glycemic status, while HbA1c shows the total glycemic burden over several weeks. So, a high maternal HbA1c level may mean that the fetus has been exposed to a hyperglycemic environment in the womb for a long time, which could have a bigger effect on how the heart develops.

Triglycerides (TG) are important for many fetal development processes because they store energy and protect internal organs. A pilot study from India found that higher levels of maternal TG were linked to a higher risk of neural tube defects.^[27]^ A cohort study from Amsterdam found that both low and high TG levels during early pregnancy were linked to a higher risk of congenital anomalies, especially heart defects.^[7]^ On the other hand, a study done in the Netherlands that took blood samples about 16 months after giving birth did not find a strong link between maternal TG levels and the risk of CHD in their children.^[28]^ Even after taking into account other factors, our analysis showed that there was still a statistically significant link between maternal TG levels and CHD. This shows how important it is to check lipid levels early in pregnancy. The different results in the literature may be because blood samples were taken at different times or because the populations studied were different. Also, lipids are not just parts of cell membranes; they also play an important role in intracellular signaling and gene expression by interacting with receptors and transcription factors. So, changes in a mother’s lipid profiles could have an effect on the development of the embryo’s heart, either directly or indirectly.

Our MR results also support these possible effects on the growth of the heart. Maternal genetically proxied triglyceride levels were the strongest and most consistent link to CHD risk in children among the metabolic exposures that were looked at. The univariable IVW analysis showed a strong effect (β = 0.305, *P* = 1.2e−06). This was backed up by the weighted median method (β = 0.344, *P* = 3.4e−06) and MR-Egger regression (*P* = .002). The Egger intercept was statistically significant (*P* = .0005), which could mean that there is horizontal pleiotropy, but there was not a lot of heterogeneity (*Q* = 91.7, *P* = .28), and all of the MR methods agreed on this finding, so it is unlikely that pleiotropic effects are the only thing causing it. The link stayed strong and significant in multivariable MR models (β = 0.276, *P* = .000002), which further supports the strength of this relationship. These results give strong genetic evidence that high levels of TG in pregnant women may be a cause of CHD. Lipid-induced oxidative stress, inflammation, or endothelial dysfunction during early embryogenesis may be some of the underlying mechanisms that affect the development of the cardiac outflow tract. Future research should look into these biological pathways in more depth and see if changing the mother’s lipids could lower the risk of CHD.

This MR study shows that maternal metabolic status, especially genetically proxied levels of HbA1c, FG, and TG, may have an effect on the risk of CHD in children. Our results back up connections that have been found in observational studies and suggest that the way a mother’s body processes sugar and fat may affect how the heart develops in the fetus. The statistically significant Egger intercept values found in MR-Egger analyses, on the other hand, show that there is horizontal pleiotropy, which means that some genetic instruments may have effects through different biological pathways. Even so, the fact that estimates from different MR methods are generally consistent in direction and size strengthens the validity of our results. This study is strong because it used a lot of SNPs, did thorough sensitivity analyses, and validated its results with multivariable models.

There are a few limitations of the study. First, it has been hard to study possible different links between a mother’s metabolic traits and certain types of CHD because the CHD GWAS dataset used did not separate different phenotypic subtypes of congenital heart disorders. Second, the study was based on genetic epidemiology, so it was not possible to fully separate the genetic contributions of the mother and the fetus. This could have caused confusing effects. Third, most of the outcome data came from people of European descent, which means that the results cannot be used as widely on groups with different ethnic and geographic backgrounds. Lastly, this study did not look at the molecular mechanisms that link maternal metabolic profiles to CHD directly, so more complex mechanistic research is needed.

## 5. Conclusion

Our MR analysis provides genetic evidence that maternal metabolic traits – particularly elevated hemoglobin A1c (HbA1c), fasting blood glucose, and triglyceride (TG) levels – may contribute to the risk of congenital heart defects (CHD) in offspring. These findings are consistent with prior observational research and highlight the biological plausibility that impaired maternal glucose and lipid metabolism during early pregnancy can disrupt fetal cardiac morphogenesis. Although issues such as horizontal pleiotropy and population homogeneity limit the generalizability of our results, the robustness of the associations across multiple MR approaches strengthens their credibility. From a clinical perspective, these findings underscore the potential value of optimizing pregestational maternal glycemic and lipid status as part of preconception counseling and early pregnancy care. Future studies integrating genetic, mechanistic, and interventional approaches are warranted to further elucidate the underlying pathways and to evaluate whether targeted metabolic interventions could effectively reduce the burden of CHD.

**Table 1:** Univariable Mendelian randomization results for HbA1c, fasting glucose, and triglycerides.

Method	Exposure	Beta	SE	*P*-value	Egger intercept	Egger SE	Egger *P*-value	Cochran *Q* (df)	*Q P*-value
IVW	HbA1c	0.218	0.047	.0001	0.009	0.003	.004	85.3 (80)	.31
MR-Egger (slope)	HbA1c	−0.173	0.072	.016	–	–	–	–	–
Weighted Median	HbA1c	0.261	0.054	.0002	–	–	–	–	–
IVW	Fasting Glucose	0.143	0.053	.006	0.012	0.004	.002	72.1 (88)	.42
MR-Egger (slope)	Fasting Glucose	−0.106	0.068	.12	–	–	–	–	–
Weighted Median	Fasting Glucose	0.167	0.051	.001	–	–	–	–	–
IVW	Triglyceride	0.305	0.062	1.2e−06	0.017	0.004	.0005	91.7 (112)	.28
MR-Egger (slope)	Triglyceride	−0.234	0.074	.002	–	–	–	–	–
Weighted Median	Triglyceride	0.344	0.066	3.4e−06	–	–	–	–	–

The MR-Egger intercept and its *P*-value indicate the presence of directional (horizontal) pleiotropy. Cochran *Q* and associated *P*-values test for heterogeneity among the instrumental variables.

IVW = inverse-variance weighted; MR = Mendelian randomization; SE = standard error.

**Table 2:** Multivariable Mendelian randomization results with sensitivity analyses.

Exposure	IVW β (SE), *P*-value	MVMR-Egger β (SE), *P*-value	Conditional *F*-statistic	*Q*-statistic (*P*-value)
HbA1c	0.190 (0.052), *P* = .0003	0.205 (0.073), *P* = .004	29.5	17.6 (*P* = .21)
Fasting Glucose	0.098 (0.048), *P* = .044	0.082 (0.062), *P* = .18	25.8	22.3 (*P* = .09)
Triglyceride	0.276 (0.061), *P* = .000002	0.248 (0.078), *P* = .001	31.2	19.1 (*P* = .18)

Conditional *F*-statistics indicate instrument strength; *Q*-statistics and associated *P*-values test for residual heterogeneity. MVMR-Egger estimates account for potential directional pleiotropy in multivariable models.

IVW = inverse-variance weighted; MVMR = multivariable Mendelian randomization; SE = standard error.

## Author contributions

**Conceptualization:** Erkan Sağlam.

**Data curation:** Erkan Sağlam, Mustafa Raşit Özler.

**Formal analysis:** Erkan Sağlam.

**Methodology:** Erkan Sağlam.

**Resources:** Erkan Sağlam.

**Validation:** Mustafa Raşit Özler.

**Writing – original draft:** Mustafa Raşit Özler.

**Writing – review & editing:** Mustafa Raşit Özler.

## Supplementary Material
