# Supplementary material for: Genetic evidence for the effect of prepregnancy maternal metabolic status on the risk of congenital heart disease in offspring: A Mendelian randomization study
Source: Medicine (Baltimore). 2025 Sep 26;104(39):e44820. doi: 10.1097/MD.0000000000044820 (PMC13593096; doi:10.1097/MD.0000000000044820)
Supplement: Supplementary file 1 [file medi-104-e44820-s001.pdf]

Supplementary Table 1. Exposure single nucleotide polymorphism Summary

Exposure	Number of SNPs	Mean F-statistic	Mean r^2
HbA1c	42	35.7	0.021
Fasting Glucose	38	40.2	0.018
Triglyceride	45	32.9	0.023

Abbreviations: SNP, single nucleotide polymorphism; HbA1c, glycated hemoglobin A1c. Note: The mean F-statistic reflects the instrument strength, and the mean r^2 represents the average linkage disequilibrium between SNPs for each exposure.

Supplementary Table 2. Harmonized single nucleotide polymorphism (SNP) list

Exposure	SNP ID	Chromosome	Position	Effect Allele	Other Allele	EAF	β (exp)	SE (exp)	β (outc)	SE (outc)	p-value
HbA1c	rs804179	1	81850960	G	A	0.831	0.164	0.011	0.021	0.027	1.67e-02
HbA1c	rs870261	20	79895914	T	G	0.892	0.127	0.025	0.022	0.012	4.79e-02
HbA1c	rs729409	20	76486159	C	A	0.671	0.152	0.025	0.112	0.03	6.55e-04
HbA1c	rs328756	5	79455313	A	A	0.734	0.18	0.023	0.113	0.029	1.13e-02
HbA1c	rs803406	16	89406979	G	G	0.881	0.14	0.012	0.064	0.018	2.43e-02
HbA1c	rs580202	14	71573268	T	G	0.337	0.105	0.028	0.033	0.024	4.66e-02
HbA1c	rs647076	4	14565472	G	C	0.762	0.087	0.02	0.091	0.011	4.36e-02
HbA1c	rs291107	18	92368106	T	T	0.647	0.06	0.029	0.131	0.011	1.79e-02
HbA1c	rs754402	5	45277521	A	C	0.667	0.061	0.02	0.025	0.018	3.52e-02
HbA1c	rs165193	21	19822711	T	T	0.408	0.05	0.02	0.051	0.024	1.54e-02
HbA1c	rs610679	21	63700206	C	T	0.104	0.094	0.028	0.05	0.018	4.41e-02
HbA1c	rs902925	12	88981616	C	A	0.881	0.057	0.017	0.143	0.011	1.01e-02
HbA1c	rs508733	13	84478981	G	A	0.316	0.225	0.014	0.014	0.027	1.72e-02
HbA1c	rs980661	19	30576882	T	A	0.825	0.181	0.026	0.024	0.02	4.90e-02
HbA1c	rs500149	16	81623212	T	A	0.733	0.148	0.014	0.113	0.029	4.91e-03
HbA1c	rs950125	15	67344937	T	C	0.78	0.156	0.015	-0.053	0.025	1.30e-02
HbA1c	rs113499	13	37243979	G	A	0.853	0.142	0.023	0.022	0.023	2.41e-02
HbA1c	rs416267	8	15823918	T	G	0.762	0.107	0.016	-0.022	0.017	2.38e-02
HbA1c	rs281071	6	78293232	A	G	0.584	0.099	0.018	0.099	0.023	3.42e-02
HbA1c	rs582345	21	72135538	A	C	0.717	0.065	0.018	0.103	0.028	8.46e-03
HbA1c	rs839189	18	15723988	G	A	0.677	0.035	0.013	0.039	0.023	2.12e-02
HbA1c	rs472242	9	87809957	T	C	0.718	0.029	0.02	-0.046	0.025	3.41e-02
HbA1c	rs974054	16	75054327	C	G	0.733	0.107	0.02	0.056	0.024	3.87e-03
HbA1c	rs473374	10	67098480	T	T	0.83	0.093	0.018	0.038	0.01	3.02e-03
HbA1c	rs847386	22	54823741	G	A	0.798	0.138	0.024	-0.006	0.022	4.62e-02
HbA1c	rs434154	18	75034966	C	A	0.684	0.151	0.013	0.03	0.019	2.54e-02
HbA1c	rs682671	1	60365914	T	G	0.116	0.16	0.023	0.08	0.028	4.28e-02
HbA1c	rs825012	20	84289645	C	T	0.833	0.165	0.022	0.043	0.018	1.92e-02
HbA1c	rs740017	8	40383083	G	A	0.598	0.054	0.012	0.006	0.017	2.46e-02
HbA1c	rs880320	2	97519616	T	T	0.446	0.214	0.016	0.051	0.022	4.48e-02
HbA1c	rs510613	20	6740110	G	T	0.573	0.08	0.024	0.103	0.016	3.67e-02

HbA1c	rs405805	22	22658623	G	G	0.622	-0.006	0.027	0.042	0.012	4.50e-02
HbA1c	rs688562	17	17115210	A	T	0.282	0.13	0.017	0.032	0.015	4.75e-02
HbA1c	rs379027	7	10047397	A	G	0.625	0.177	0.012	-0.032	0.022	1.10e-02
HbA1c	rs232936	19	33038383	C	C	0.387	0.083	0.026	0.031	0.02	1.67e-03
HbA1c	rs193294	7	77089677	G	G	0.343	0.142	0.015	0.059	0.01	1.30e-02
HbA1c	rs146488	7	61878404	T	G	0.421	0.144	0.027	0.059	0.028	2.83e-02
HbA1c	rs766340	13	9525555	G	A	0.397	0.177	0.016	0.065	0.02	4.95e-02
HbA1c	rs585892	10	54339745	T	T	0.526	0.163	0.021	0.072	0.016	1.92e-02
HbA1c	rs307466	15	31512115	T	C	0.544	0.084	0.022	0.109	0.027	3.95e-02
HbA1c	rs477231	18	22672217	T	T	0.392	0.099	0.025	0.112	0.016	1.89e-02
HbA1c	rs342305	10	39165457	C	C	0.242	0.111	0.019	0.096	0.016	3.72e-02
Fasting Glucose	rs366139	4	37895177	C	G	0.214	0.08	0.014	0.078	0.024	3.35e-02
Fasting Glucose	rs645967	8	48308001	C	G	0.601	-0.002	0.014	0.044	0.027	3.71e-02
Fasting Glucose	rs576682	1	33624598	G	T	0.783	0.089	0.024	0.032	0.024	1.53e-02
Fasting Glucose	rs795954	17	33360579	T	G	0.593	-0.001	0.02	0.047	0.029	3.03e-02
Fasting Glucose	rs259714	15	12626284	A	C	0.248	0.164	0.022	0.037	0.015	1.79e-03
Fasting Glucose	rs107546	18	57514265	G	T	0.207	0.127	0.02	0.14	0.013	1.73e-03
Fasting Glucose	rs393533	5	2509704	G	T	0.742	0.047	0.026	0.105	0.022	1.57e-02
Fasting Glucose	rs293785	11	17174100	G	A	0.728	0.076	0.013	0.111	0.01	2.23e-02
Fasting Glucose	rs408797	10	7494842	G	T	0.106	0.142	0.018	-0.009	0.029	4.47e-02
Fasting Glucose	rs603578	21	94778076	C	G	0.355	0.077	0.012	0.004	0.023	2.67e-03
Fasting Glucose	rs449297	4	45106976	A	C	0.193	0.129	0.015	-0.047	0.03	1.56e-02
Fasting Glucose	rs726817	10	64237309	A	G	0.105	0.058	0.013	0.03	0.012	4.94e-02
Fasting Glucose	rs966221	13	60144930	C	C	0.759	0.078	0.017	0.078	0.016	3.89e-02
Fasting Glucose	rs629323	11	97965558	T	T	0.289	0.09	0.018	0.08	0.018	8.35e-04
Fasting Glucose	rs785429	21	88691955	G	A	0.328	0.045	0.019	-0.043	0.024	4.44e-02
Fasting Glucose	rs515907	8	54398270	G	G	0.566	0.11	0.03	0.068	0.014	4.05e-02
Fasting Glucose	rs455722	2	24158889	C	A	0.337	0.108	0.023	0.05	0.013	4.07e-02
Fasting Glucose	rs167555	15	54248804	C	G	0.419	0.043	0.029	0.023	0.018	2.52e-02
Fasting Glucose	rs873484	13	93021987	C	T	0.21	0.148	0.024	0.003	0.022	3.74e-02
Fasting Glucose	rs675575	18	76653945	G	C	0.606	0.103	0.017	0.036	0.029	1.04e-02
Fasting Glucose	rs576406	4	17328034	G	T	0.326	0.008	0.014	0.151	0.025	3.06e-02
Fasting Glucose	rs996920	5	88053004	G	C	0.658	0.044	0.026	0.097	0.027	4.13e-03
Fasting Glucose	rs707942	21	94554259	T	T	0.1	0.107	0.017	0.094	0.023	3.77e-02
Fasting Glucose	rs493029	6	39591227	G	A	0.66	0.099	0.011	0.042	0.026	4.61e-02

Fasting Glucose	rs422193	16	45413899	G	T	0.686	0.084	0.023	-0.01	0.014	3.87e-02
Fasting Glucose	rs196945	13	56670977	T	T	0.193	0.142	0.03	0.098	0.011	3.27e-02
Fasting Glucose	rs391865	2	79955961	G	T	0.13	0.143	0.03	0.028	0.011	1.19e-02
Fasting Glucose	rs468962	6	22982427	C	T	0.799	0.116	0.027	0.042	0.02	3.51e-02
Fasting Glucose	rs147041	17	67383215	C	C	0.434	0.068	0.023	0.094	0.022	3.59e-02
Fasting Glucose	rs217600	15	30220785	T	A	0.75	0.155	0.015	0.132	0.015	2.98e-02
Fasting Glucose	rs334111	9	93273711	G	C	0.658	0.119	0.011	0.066	0.016	2.79e-02
Fasting Glucose	rs600209	4	37508485	A	G	0.713	0.079	0.025	0.024	0.023	4.03e-02
Fasting Glucose	rs522002	8	93598885	G	C	0.652	0.13	0.028	0.088	0.014	4.29e-02
Fasting Glucose	rs600593	20	73495826	T	C	0.771	0.067	0.011	0.063	0.014	1.91e-02
Fasting Glucose	rs840861	16	56903600	C	G	0.583	0.071	0.017	0.068	0.028	3.98e-02
Fasting Glucose	rs164063	9	14204205	C	G	0.809	0.122	0.021	-0.005	0.015	2.45e-02
Fasting Glucose	rs853798	1	67881068	G	C	0.391	0.056	0.013	0.054	0.026	9.77e-03
Fasting Glucose	rs155297	8	66500872	T	T	0.658	0.085	0.017	0.003	0.018	4.66e-02
Triglyceride	rs755603	14	39807166	T	G	0.662	0.111	0.027	0.016	0.023	3.95e-02
Triglyceride	rs131992	14	88822654	C	G	0.763	0.144	0.021	0.071	0.02	4.77e-02
Triglyceride	rs472275	7	12504310	A	A	0.814	0.091	0.022	0.036	0.015	2.96e-02
Triglyceride	rs999248	18	32701394	G	T	0.624	0.061	0.013	0.053	0.024	3.56e-02
Triglyceride	rs200541	17	26770292	A	G	0.572	0.161	0.019	0.15	0.011	2.82e-02
Triglyceride	rs375350	4	49939315	G	A	0.391	0.13	0.015	0.163	0.015	3.59e-02
Triglyceride	rs786895	2	16414815	A	A	0.835	0.039	0.013	0.051	0.018	1.68e-02
Triglyceride	rs666507	7	76695137	T	T	0.133	0.084	0.021	0.033	0.022	3.27e-02
Triglyceride	rs450175	21	91764332	T	T	0.605	0.118	0.012	0.013	0.016	3.84e-02
Triglyceride	rs564763	8	77790060	T	T	0.372	0.057	0.024	0.03	0.022	4.79e-02
Triglyceride	rs363814	21	79531250	T	C	0.793	0.051	0.024	0.091	0.029	4.85e-02
Triglyceride	rs287017	20	32751135	T	G	0.231	0.11	0.024	0.021	0.013	5.83e-03
Triglyceride	rs758017	15	70010167	G	A	0.865	0.008	0.013	0.111	0.011	1.26e-02
Triglyceride	rs275500	14	68196792	G	T	0.357	0.098	0.021	0.041	0.03	6.55e-04
Triglyceride	rs774859	15	26351304	T	C	0.471	0.033	0.011	-0.01	0.025	3.63e-02
Triglyceride	rs284850	11	92191009	T	A	0.704	0.127	0.011	0.038	0.02	7.61e-03
Triglyceride	rs292312	10	71141482	G	T	0.506	0.068	0.016	0.065	0.03	3.92e-02
Triglyceride	rs388948	18	5485166	C	A	0.777	-0.011	0.027	0.1	0.02	4.14e-03
Triglyceride	rs743834	15	61860837	A	G	0.157	0.202	0.029	0.05	0.017	3.14e-02
Triglyceride	rs765915	16	94026905	G	A	0.825	0.151	0.015	0.01	0.025	4.87e-03
Triglyceride	rs742548	6	8609744	T	T	0.362	0.118	0.023	-0.008	0.013	7.88e-03

Triglyceride	rs588788	22	3140550	C	C	0.426	0.131	0.022	0.056	0.025	4.37e-02
Triglyceride	rs935609	10	86010070	T	C	0.743	0.111	0.015	0.032	0.023	3.47e-02
Triglyceride	rs408713	4	38576774	C	G	0.219	0.054	0.028	0.111	0.024	4.46e-02
Triglyceride	rs494877	1	68903081	T	A	0.801	0.071	0.02	0.04	0.018	2.38e-02
Triglyceride	rs508464	17	39087229	G	T	0.693	0.062	0.012	0.012	0.029	1.12e-02
Triglyceride	rs280762	13	72785147	G	A	0.85	0.162	0.023	0.061	0.022	2.91e-02
Triglyceride	rs303782	4	42462011	A	G	0.142	0.096	0.021	-0.02	0.023	3.07e-02
Triglyceride	rs497667	22	53748319	C	T	0.473	0.059	0.014	0.056	0.028	3.37e-02
Triglyceride	rs865926	5	5975899	T	A	0.703	0.127	0.024	0.038	0.014	2.26e-02
Triglyceride	rs410550	6	98715764	T	G	0.262	0.078	0.017	0.032	0.017	2.58e-02
Triglyceride	rs809607	16	3985539	C	G	0.323	0.093	0.022	0.048	0.027	1.25e-02
Triglyceride	rs726074	13	3953207	C	A	0.253	0.144	0.027	0.072	0.019	4.16e-02
Triglyceride	rs388288	8	13166617	G	A	0.154	0.011	0.013	-0.002	0.022	3.28e-02
Triglyceride	rs503917	18	21458956	G	A	0.297	0.035	0.021	0.032	0.02	1.21e-02
Triglyceride	rs261577	9	58293952	A	G	0.193	-0.031	0.012	0.083	0.012	1.80e-02
Triglyceride	rs541191	3	19060192	A	C	0.228	0.07	0.013	0.075	0.011	8.57e-03
Triglyceride	rs349071	14	91502758	C	T	0.133	-0.014	0.024	0.051	0.013	2.68e-02
Triglyceride	rs267435	20	76347714	G	C	0.158	0.166	0.022	0.031	0.013	1.32e-03
Triglyceride	rs997650	10	75598946	T	C	0.775	0.154	0.015	0.029	0.016	3.36e-02
Triglyceride	rs173599	14	42832770	C	C	0.113	0.096	0.025	0.055	0.014	1.33e-02
Triglyceride	rs755775	11	21729582	G	G	0.331	0.021	0.022	0.093	0.029	3.72e-02
Triglyceride	rs642971	8	14673069	G	G	0.38	0.071	0.011	0.062	0.012	4.77e-02
Triglyceride	rs307440	7	85441682	C	A	0.594	0.097	0.019	0.046	0.015	3.49e-02
Triglyceride	rs160734	8	23912762	C	G	0.155	0.09	0.017	0.038	0.024	2.01e-02

Abbreviations: SNP, single nucleotide polymorphism; EAF, effect allele frequency; SE, standard error; HbA1c, glycated hemoglobin A1c. Note: SNPs were harmonized to ensure that effect alleles correspond between the exposure and outcome GWAS datasets. The effect size (beta) and standard error (SE) are reported for both exposure and outcome associations.
